# SUV39H1 downregulation induces deheterochromatinization of satellite regions and senescence after exposure to ionizing radiation

**DOI:** 10.3389/fgene.2014.00411

**Published:** 2014-11-21

**Authors:** Corinne Sidler, Dongping Li, Bo Wang, Igor Kovalchuk, Olga Kovalchuk

**Affiliations:** Department of Biological Sciences, University of LethbridgeLethbridge, AB, Canada

**Keywords:** senescence, aging, ionizing radiation, epigenetics, SUV39H1, DNA methylation

## Abstract

While the majority of cancer patients are exposed to ionizing radiation during diagnostic and therapeutic procedures, age-dependent differences in radiation sensitivity are not yet well understood. Radiation sensitivity is characterized by the appearance of side effects to radiation therapy, such as secondary malignancies, developmental deficits, and compromised immune function. However, the knowledge of the molecular mechanisms that trigger these side effects is incomplete. Here we used an *in vitro* system and showed that low-senescent normal human diploid fibroblasts (WI-38) senesce in response to 5 Gy IR, while highly senescent cultures do not show changes in cell cycle regulation and only a slight increase in the percentage of senescent cells. Our study shows that this is associated with changes in the expression of genes responsible for cell cycle progression, apoptosis, DNA repair, and aging, as well as transcriptional and epigenetic regulators. Furthermore, we propose a role of the downregulation of *SUV39H1* expression, a histone methyltransferase that specifically trimethylates H3K9, and the corresponding reduction in H3K9me3 levels in the establishment of IR-induced senescence.

## Introduction

Age is a major risk factor for cancer. However, while the majority of cancer patients undergo diagnostic and therapeutic procedures involving ionizing radiation (IR), age-dependent differences in radiation sensitivity have not been well understood. Follow-up studies of patients that have received radiation therapy and studies on survivors of the atomic bombings in Japan have shown the wide variety of side effects associated with those exposures, including the risk of developing secondary cancers (Kleinerman, 2006), compromised immune function (Yamaoka et al., 2004), and developmental deficits (Krasin et al., 2010).

Many of these side effects are probably caused by the exposure of non-tumor tissue to IR, as many of the currently available follow-up studies are based on information on patients that were subjected to less precisely targeted treatment. However, even with current three-dimensional irradiation strategies, residual exposure of healthy tissues to scattered radiation cannot be completely avoided (Joosten et al., 2013). In pediatric patients, the effects of IR on growth and development are a concern (Krasin et al., 2010); in adult patients, the effects of IR on organ function is a greater concern, as the repair capacity in aging organs may be more limited (Krishnamurthy et al., 2006).

Aging is associated with various molecular changes that may affect the outcome of the response to IR. Aging tissues have been shown to accumulate senescent cells (Dimri et al., 1995), which exhibit shortened telomeres (Harley et al., 1990); oxidative damage and oxidative stress (Wolf et al., 2002); decreased fidelity and efficiency of DNA double-strand break repair (Seluanov et al., 2004); altered chromatin structure (Scaffidi and Misteli, 2006; O'sullivan et al., 2010), including the formation of senescence-associated heterochromatin foci at the expense of constitutive heterochromatin (Narita et al., 2003); and a pro-inflammatory secretion profile (Rodier et al., 2009).

On the other hand, the response to IR is characterized by the activation of an ataxia telangiectasia mutated (ATM)-dependent DNA damage checkpoint (Bakkenist and Kastan, 2003), which enables DNA repair. Thus, cells recover from the DNA damage checkpoint upon successful DNA repair and removal of the γH2AX DNA damage signal (Keogh et al., 2006). A failure to repair the DNA damage associated with prolonged G2 arrest may result in p53-dependent apoptosis (Lowe et al., 1993), checkpoint recovery followed by mitotic catastrophe (Castedo et al., 2004), or slippage into the G1 phase and undergo senescence (Ye et al., 2013). The extent to which cells become senescent or undergo apoptosis depends on the cumulative dose of IR the cells were exposed to Noda et al. (2012).

Further, the physiology of the response to IR also seems to depend on the state of senescence: senescent cells are more resistant to radiation-induced cell death than their dividing progenitors (Latella et al., 2004). However, the molecular mechanisms underlying the differences between the dividing and senescent cells in response to IR are not yet well understood.

Therefore, in this study we compared the physiological response of WI-38 cells at three different stages of senescence to IR, and evaluated the associated changes in gene expression profiles. Here, we show that the exposure to 5 Gy of IR resulted in extensive changes to the gene expression profile. The most prominent physiological response to IR was observed in young cultures, which predominantly underwent senescence. Here we propose a role for *SUV39H1* in this establishment of IR-induced senescence.

## Materials and methods

### Cell culture

WI-38 human fetal lung fibroblasts (ATCC, CCL-75TM, LOT 58110309) were maintained in HyClone minimum essential medium (MEM) Alpha Modification (ThermoScientific) containing 10% (v/v) fetal bovine serum (FBS; Gibco) in a humidified Forma Steri-Cycle CO_2_ Incubator (ThermoScientific) containing 6% CO_2_ at 37°C. WI-38 cells have a replicative limit of 50 ± 10 population doublings (PDs). In this study, cultures of three different PDs were used—38 (young), 47 (intermediate), or 54 (old), as previously described (Sidler et al., 2014). The PD number of a culture is the sum of all ΔPD = log_2_(n_f_/n_i_) for each passage, where n_f_ is the final number of cells in a passage and n_i_ is the initial number of cells inoculated.

### X-ray exposure

The cells were irradiated with doses of 0, 0.5, and 5 Gy of 90 kVp and 5 mA at 0.93 Gy/min (Faxitron X-ray LLC, Model RX-650). The samples were collected at 48 h post-exposure unless stated otherwise. Samples for RNA or DNA isolation were snap-frozen in liquid nitrogen and stored at −80°C until further processing. Samples for protein isolation or flow cytometry were processed as described in the subsequent sections.

### Gene expression profiling

For the determination of gene expression profiles, RNA was extracted from 2 to 3 samples per group using the TRIzol® Reagent (Invitrogen) according to manufacturer's instructions. Total RNA was quantified using NanoDrop2000c (ThermoScientific), and the RNA integrity was determined using a 2100 BioAnalyzer (Agilent). Illumina® HumanHT12-v4 Gene Expression BeadChips were used to determine the transcript levels using 47231 unique probes. Illumina® GenomeStudio software was used for differential expression analysis using an Illumina custom model with an FDR of 0.05. Only genes for which the differential expression analysis was significant at a level of *p* < 0.05 and had a log2 fold change of less than −0.4 or greater than 0.4 were considered for further analysis.

### DNA methylation profiling

For DNA methylation profiling, genomic DNA (gDNA) was isolated from cells using the DNeasy® Blood and Tissue Kit (Qiagen). gDNA was recovered in 100 μL nuclease-free water. The gDNA was then treated with ribonuclease A (0.1 mg/ml final concentration) for 1 h at 37°C and purified by phenol:chloroform extraction. DNA was precipitated by adding 3 M of sodium acetate (pH 5.2) in the ratio 1:5 and 100% ethanol in the ratio 5:1. DNA pellets were dissolved in 50 μL nuclease-free water and quantified using NanoDrop 2000c (ThermoScientific). Illumina® HumanMethylation27 BeadChips were used to determine the methylation levels of more than 28,000 unique CpG sites. Among the BeadChip probes, most of the CpG sites detected were located in the promoter regions of genes, with a slight overrepresentation of cancer genes. In order to determine the relative methylation levels of specific sites among the different treatment groups, beta values (percentage methylation of a specific CpG site in a specific sample) were determined using Illumina® GenomeStudio software. Differential DNA methylation analyses were performed using an Illumina custom model, which produced diff scores as a measure of significance. Diff scores of −13/13 were set as cutoff values for significance.

### Functional classification of genes

Online software and databases were used for the functional classification of differentially expressed genes or differentially methylated CpG sites: FunNet Transcriptional Networks Analysis (www.funnet.info), DAVID Bioninformatics Resources 6.7 (Huang da et al., 2009b,a), and the Genecards database (http://www.genecards.org) (Safran et al., 2010).

### Quantitative real-time PCR (qRT-PCR)

Transcription levels of the selected genes were confirmed using qRT-PCR. The RNA samples were treated with DNAse I using an Illustra RNAspin Mini Prep (GE Healthcare) to remove any gDNA contamination. The RNA was quantified by NanoDrop2000c, and 500 ng of RNA was used for cDNA synthesis using the iScript™ Select cDNA synthesis kit (BioRad). qRT-PCR reactions were set up using the SsoFast™ EvaGreen® Supermix (BioRad) together with primers specific for the target sequences of interest (S1) and analyzed on a C1000^TM^ Thermo Cycler equipped with a CFX96^TM^ Real-Time System (BioRad). PCR conditions were chosen according to the SSoFast guidelines with the annealing temperatures specified for primer pairs (S2).

Each experiment included three biological replicates and two technical replicates per treatment. The *HPRT1, RPL13A*, and *YWHAZ* housekeeping genes were used for the normalization and calculation of transcript levels using qbase^PLUS^ (Vandesompele et al., 2002).

### Western blotting

Total protein extracts were prepared by sonicating cells harvested from two 10-cm cell culture dishes per sample in 100 μL of cold 1% sodium dodecyl sulfate (SDS) containing protease inhibitor (Roche). The protein amounts were quantified using Bradford assays (BioRad) and measuring the absorbance at 595 nm using NanoDrop 2000c (ThermoScientific). Equal amounts of proteins per lane (10–40 μg depending on the protein of interest) were separated by SDS-polyacrylamide gel electrophoresis (PAGE) in slab gels of 6–15% polyacrylamide and transferred to polyvinylidene difluoride (PVDF) membranes (Hybond-P, Amersham Biosciences). The proteins were then incubated with primary antibody overnight at 4°C (S2), followed by incubation with a secondary horseradish peroxidase-conjugated antibody (S2). Antibody binding was detected using an enhanced chemiluminescence plus immunoblotting detection system (Amersham Biosciences). Chemiluminescence was detected using a FluorChem HD2 camera with FluorChem software (Cell Biosciences). Unaltered PVDF membranes were stained with Coomassie blue (BioRad) to confirm equal protein loading. Chemiluminescence signals were quantified using NIH ImageJ 64 software. Pixel intensities of the protein bands of interest were normalized to GAPDH pixel intensities.

### Flow cytometry analysis

#### Cell isolation

Cells from two 10-cm cell culture plates per sample were combined for each treatment. The cells were harvested by trypsinization, centrifugation at 200 *g* for 5 min at 4°C and resuspended in 1 mL Dulbecco's phosphate buffered saline (DPBS) (Lonza) before starting specific staining procedures, except in the case of senescence-associated β-galactosidase assay, for which the staining was performed in the cell culture plates.

#### Senescence-associated β-galactosidase (SA-β-GAL) assay

In order to quantify the amounts of senescent cells in response to different X-ray treatments, a flow cytometry-based fluorescent SA-β-GAL staining was employed. The staining was carried out using the method described by Debacq-Chainiaux et al. (2009) for 1.5 h at standard culture conditions (Debacq-Chainiaux et al., 2009). Three samples were analyzed for each treatment on a BD FACS Canto II cytometer (BD Biosciences). Measurements included 10,000 events per sample.

#### Detection of apoptotic, necrotic, and dead cells

A fluorescein isothiocyanate (FITC) annexin V Apoptosis Detection Kit (BD Pharmigen™) was used to detect the apoptotic, necrotic, and dead cells in the different treatment groups, according to the manufacturer's instructions. Three samples were analyzed per treatment using a BD FACS Canto II cytometer (BD Biosciences). Measurements included 10,000 events per sample.

#### Detection of proliferating cells

BrdU is a nucleotide analog that is incorporated into newly synthesized DNA; it is therefore commonly utilized in the detection of DNA synthesis. BrdU staining was performed using a BrdU flow kit (BD Pharmigen™). Cells were pulsed with 1 mM BrdU in MEM for 40 min at standard culture conditions, and then processed according to the manufacturer's manual. Three samples per treatment were analyzed on a BD FACS Canto II cytometer (BD Biosciences) and 10,000 events were detected per sample.

### SUV39H1 overexpression

The overexpression of *SUV39H1* was achieved by transfecting cells with the *pCMV6-SUV39H1* expression construct (Origene) using Lipofectamine™ 2000 (Invitrogen).

### Chromatin immunoprecipitation

For testing of p53 binding to the *SUV39H1* promoter, we performed chromatin immunoprecipitation as described previously (Nelson et al., 2006). Briefly, chromatin was cross-linked by treating cells with 1% formaldehyde for 15 min and quenching with 125 mM glycine for 5 min. Then, 12–14 million cells were sonicated in 500 μL immunoprecipitation buffer (150 mM NaCl, 50 mM Tris–HCl [pH 7.5], 5 mM EDTA, 0.5% NP-40, 1% Triton X-100, 0.5 mM phenylmethylsulfonyl fluoride (PMSF), leupeptin, and proteinase inhibitor [Roche]) to shear the DNA into 100–1000-bp fragments. Immunoprecipitation was performed using antibodies against rabbit or mouse IgG as a negative control, or rabbit anti-Acetyl-K382 p53 (Cell Signaling), or mouse anti-p53 (Santa Cruz). The DNA fragments were then isolated using 10% Chelex®100 and purified using a QIAquick® PCR purification kit.

**Figure 1:**
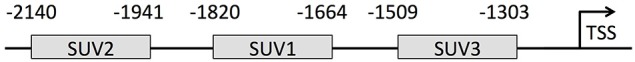
**ChIP-qPCR primers targeted to the *SUV39H1* promoter**. TSS indicates the transcription start site. Numbers indicate the primer start site upstream of the TSS.

ChIP-qRT-PCR was performed like qRT-PCR using the primers listed in S3. Figure 1 shows a representation of the location of the amplicons within the *SUV39H1* promoter. The results were calculated by averaging the 2^(−Ct)^ values of three samples per treatment and normalizing to 10% of the values obtained from the input DNA.

### Statistical analysis

Each data point is an average of three biological replicates ± standard deviation (*SD*) unless stated otherwise. Statistical significance was determined by a Student's *t*-test based on a *p*-value of less than 0.05. Statistical analysis of the gene expression and DNA methylation BeadChips is described in the according methods sections.

## Results

### Exposure to IR induces differential gene expression at all stages of senescence

**Figure 2:**
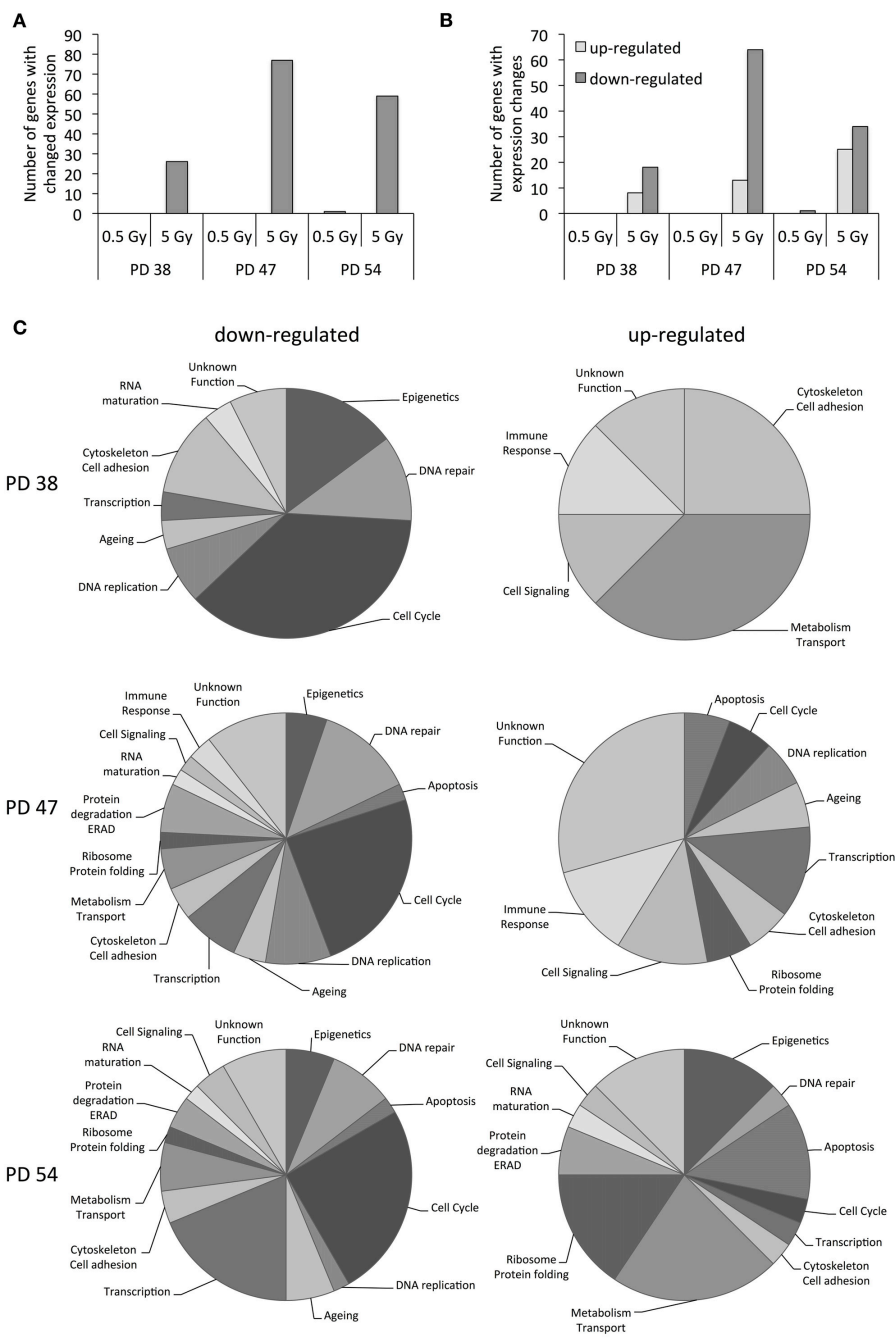
**Functional classification of gene expression results. (A)** Total number of genes affected by expression changes in response to ionizing radiation. **(B)** Numbers of up- (light gray) and downregulated (dark gray) genes in response to IR. **(C)** Functional classification of genes that were differentially expressed in response to 5 Gy of IR.

In order to better understand how cell cultures of different stages of senescence react to X-ray irradiation, we exposed cultures at three different stages of senescence to 0.5 or 5 Gy irradiation and profiled the mRNA expression patterns using Illumina® Gene Expression BeadChips. The results revealed that exposure to 5 Gy of X-ray affected gene expression profiles more strongly than exposure to 0.5 Gy (Figure 2A). Cultures with an intermediate senescence ratio (PD 47) showed the highest number of differentially expressed genes (77), followed by cultures with high senescence ratio (PD 54) with 59 differentially expressed genes and cultures with low senescence ratio (PD 38) with 26 differentially expressed genes (S4). Of all these expression changes, there was a bias toward transcription repression (Figure 2B). However, with increasing senescence ratio of the cultures, the number of genes upregulated in response to 5 Gy increased.

Functional classification was performed to understand the functional implications of the observed changes in expression. The genes related to epigenetic regulation, DNA repair, cell cycle regulation, aging, regulation of transcription, and cell adhesion were among the genes downregulated in response to 5 Gy in cells of all ages (Figure 2C). The functions that were commonly induced in response to 5 Gy included cell adhesion, metabolism, and cell signaling and in the older cultures (PD 47 and PD 54), DNA repair, apoptosis, and cell cycle regulation were also induced by irradiation (Figure 2C).

### Pre-senescent cultures induce G2/M arrest and senescence in response to X-ray irradiation

IR is known to induce cell cycle arrest at the G1/S or G2/M checkpoints (Terasima and Tolmach, 1963), senescence (Ye et al., 2013), and apoptosis (Neal and Potten, 1981; Warters, 1992). Correspondingly, we observed downregulation of cell cycle regulators in cultures of all ages and up-regulation of apoptotic genes in intermediate (PD 47), and old (PD 54) cultures. Based on these differences in gene expression, we hypothesized that the physiology of the response to X-ray may differ at different stages of senescence.

**Figure 3:**
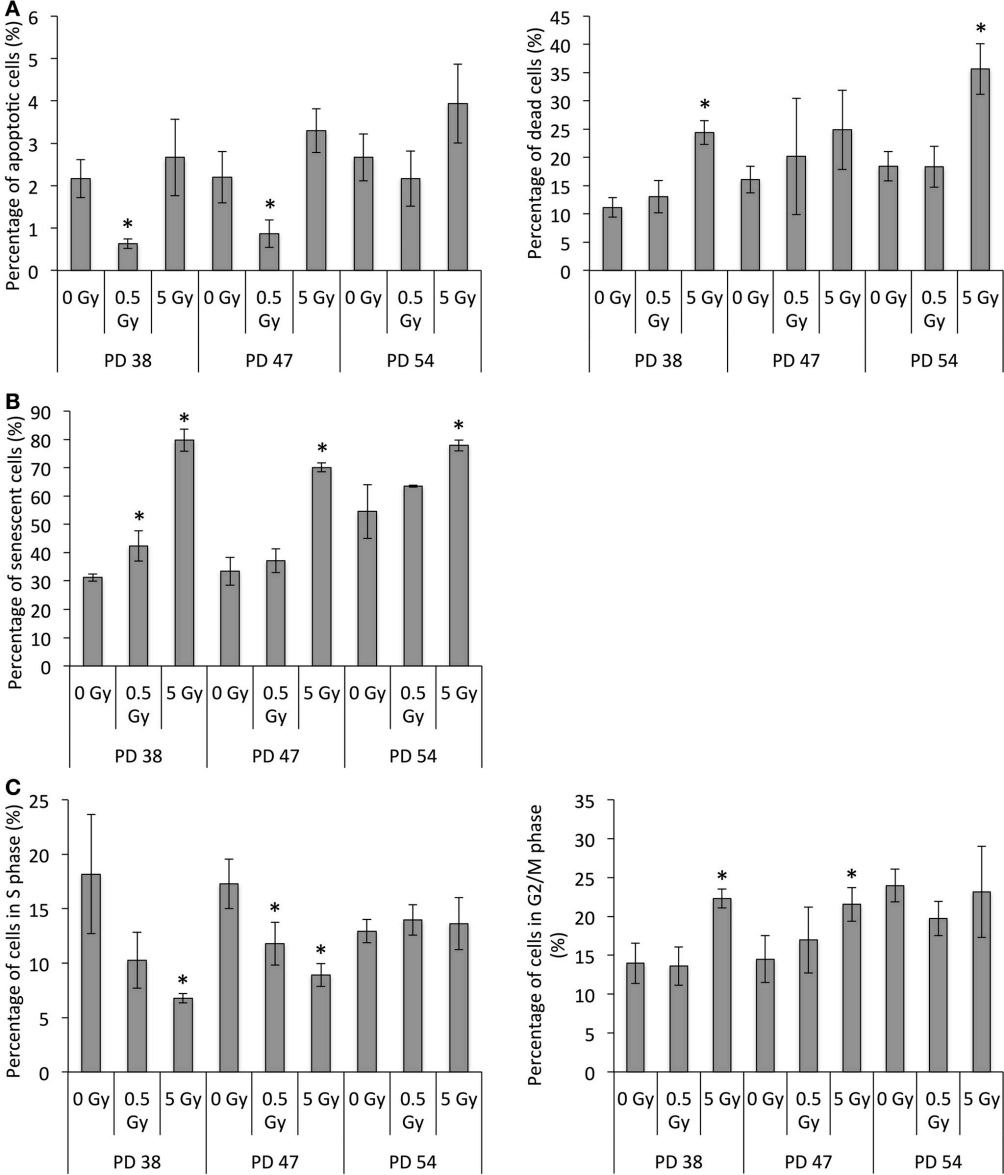
**Radiation-induced cell growth arrest, senescence, and cell death. (A)** Percentage of apoptotic (Annexin V positive) or dead (PI positive) cells as determined by annexin V/PI staining. **(B)** Percentage of senescent cells as determined by SA-β-GAL staining. **(C)** Percentage of cells in the S or G2/M phase determined by BrdU incorporation and DNA staining. Averages of three samples ± *SD*. Asterisks on bars indicate significant difference to the control of the PD level in all graphs.

To test this, we detected proliferating, senescent, and apoptotic cells in cultures exposed to 0.5 Gy or 5 Gy by using BrdU, SA-β-GAL, and annexin V/PI staining respectively. Exposure to 5 Gy resulted in an increase in apoptotic (positive for annexin V, but not PI) and dead (positive for PI staining) cells in cultures of all ages at 48 h post-exposure, although the differences were significant only for dead cells in the PD 38 and PD 54 cultures (Figure 3A, S5). Further, the number of apoptotic cells in 0.5 Gy-irradiated PD 38 cultures was significantly lower than in non-irradiated cells, while the number of dead cells was not significantly different between the two treatments.

Further, the number of senescent cells at 48 h after irradiation exposure increased in all cultures exposed to 5 Gy and in PD 38 cultures exposed to 0.5 Gy (Figure 3B, S5). The highest increase in senescence ratio was observed in PD 38 cells exposed to 5 Gy.

The analysis of cell cycle distribution based on the BrdU pulse and DNA staining at 48 h after exposure revealed a significant decrease in S-phase cells in the PD 38 and PD 47 groups with increasing dose of IR (Figure 3C, S5). This is in line with the increased induction of senescence in response to 5 Gy in PD 38 and PD 47 cells. In addition, this may also indicate that exposure to 0.5 and 5 Gy can induce cell cycle arrest. Accordingly, exposure to 5 Gy resulted in an increased cell population in the G2/M phase of the cell cycle in PD 38 and PD 47 cells. However, no radiation-dependent changes in cell cycle distribution could be detected in PD 54 cultures (Figure 3C, S5).

In summary, the surviving fraction of cells in the young group either enters cell cycle arrest or senescence and the surviving fraction in the more senescent groups is smaller than that in younger cultures and they exhibit senescence rather than cell cycle arrest.

### Changes in cell cycle regulation and age-related gene expression correlate with radiation-induced senescence

The increase in senescent cells in the irradiated cell cultures of all PD levels correlated with the changes in age-related gene expression. A comparison of the obtained gene expression profiles with microarray signatures from the Human Ageing Genomic Resources (de Magalhaes et al., 2009) and information from Genecards (Safran et al., 2010) showed that five senescence-associated genes were differentially expressed in one or more experimental groups (Table 1).

**Table 1:** **Senescence-associated gene expression**.

**Gene**	**Involvement in ageing**	**PD 38**		**PD 47**		**PD 54**	
		**0.5 Gy**	**5 Gy**	**0.5 Gy**	**5 Gy**	**0.5 Gy**	**5 Gy**
*COL3A1*	Downregulated with age					0.30	
*CTGF*	Upregulated with senescence				0.43		
*MNT*	May induce senescence						−0.44
*SUV39H1*	H3K9 methylation-dependent induction of senescence		−0.42		−0.43		
*TIMP3*	May be involved in senescence		0.29		0.37		

*Entries for genes that were upregulated/downregulated with age are based on microarray signatures from Human Ageing Genomic Resources (de Magalhaes et al., 2009). Other gene information is based on Genecards (http://www.genecards.org/) (Safran et al., 2010). Numbers indicate log2-fold changes compared to the controls*.

The expression levels of a senescence-associated gene (*COL3A1*) and a cell cycle regulator (*UBE2C*) were confirmed by qRT-PCR (S6).

### Reduced SUV39H1 expression in irradiated cultures correlates with reduced H3K9 trimethylation

While none of the differentially expressed transcription factors showed an enrichment of its target genes among the differentially expressed genes, changes in epigenetic regulation may underlie some of the observed changes in gene expression patterns. As both *SUV39H1*, a histone methyltransferase that specifically trimethylates H3K9, and *CBX5*, which binds to trimethylated H3K9 (H3K9me3) and mediates its repressive function, were consistently downregulated in PD 38 and PD 47 cells exposed to 5 Gy irradiation (S4), we hypothesized that changes in the chromatin structure may be involved in this response.

**Figure 4:**
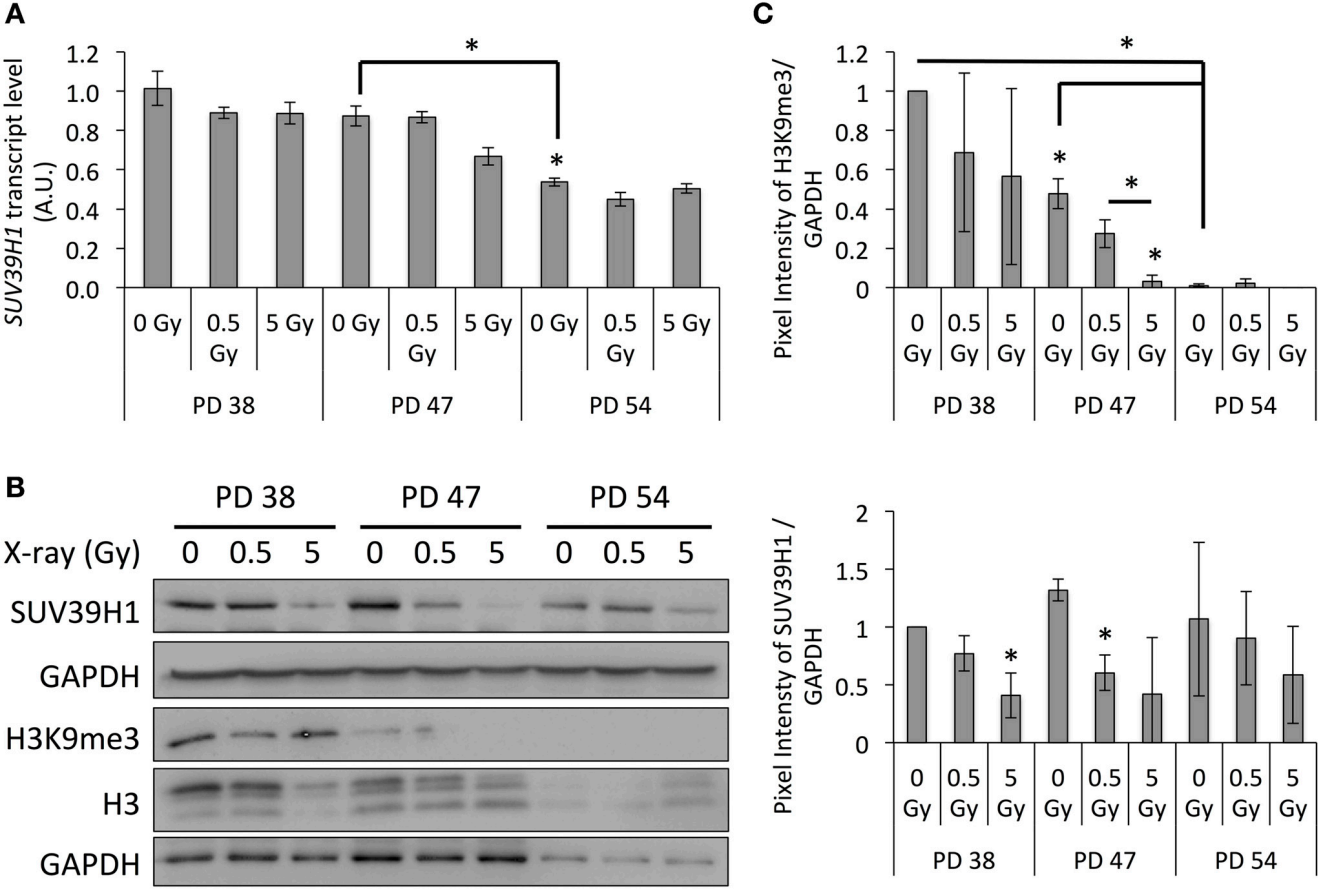
**Regulation of SUV39H1 expression and correlation with H3K9me3 levels in response to IR. (A)** *SUV39H1* transcript levels normalized to PD 38 0 Gy (Arbitrary units, A.U.). Averages of three biological repeats with two technical replicas each. Error bars indicate error progression of SDs. **(B)** Western blots images with respective GAPDH loading controls. **(C)** Western blot quantifications of the SUV39H1 and H3K9me3 protein levels normalized to pixel intensities in PD 38 0 Gy. Averages of three biological replicas ± *SD*. ^*^p < 0.05.

When measuring the *SUV39H1* expression at the mRNA and protein levels by qRT-PCR and Western blot analysis respectively, we detected a significant senescence-dependent decrease at both the mRNA and protein levels (Figures 4A–C). In addition, results of the Western blot analysis revealed that the SUV39H1 levels were significantly decreased in the PD 38 and PD 47 groups following IR exposure (Figures 4B,C). This finding was further correlated with a significant decrease in the H3K9me3 levels in response to senescence and IR exposure in the PD 47 group (Figures 4B,C). Interestingly, similar observations were made for the global histone 3 levels, although this reduction was less severe than the reduction in H3K9me3 levels (Figures 4B,C). Taken together, these results may suggest that the downregulation of SUV39H1 in response to IR results in a loss of heterochromatin, which may result in altered gene expression or genomic instability.

**Figure 5:**
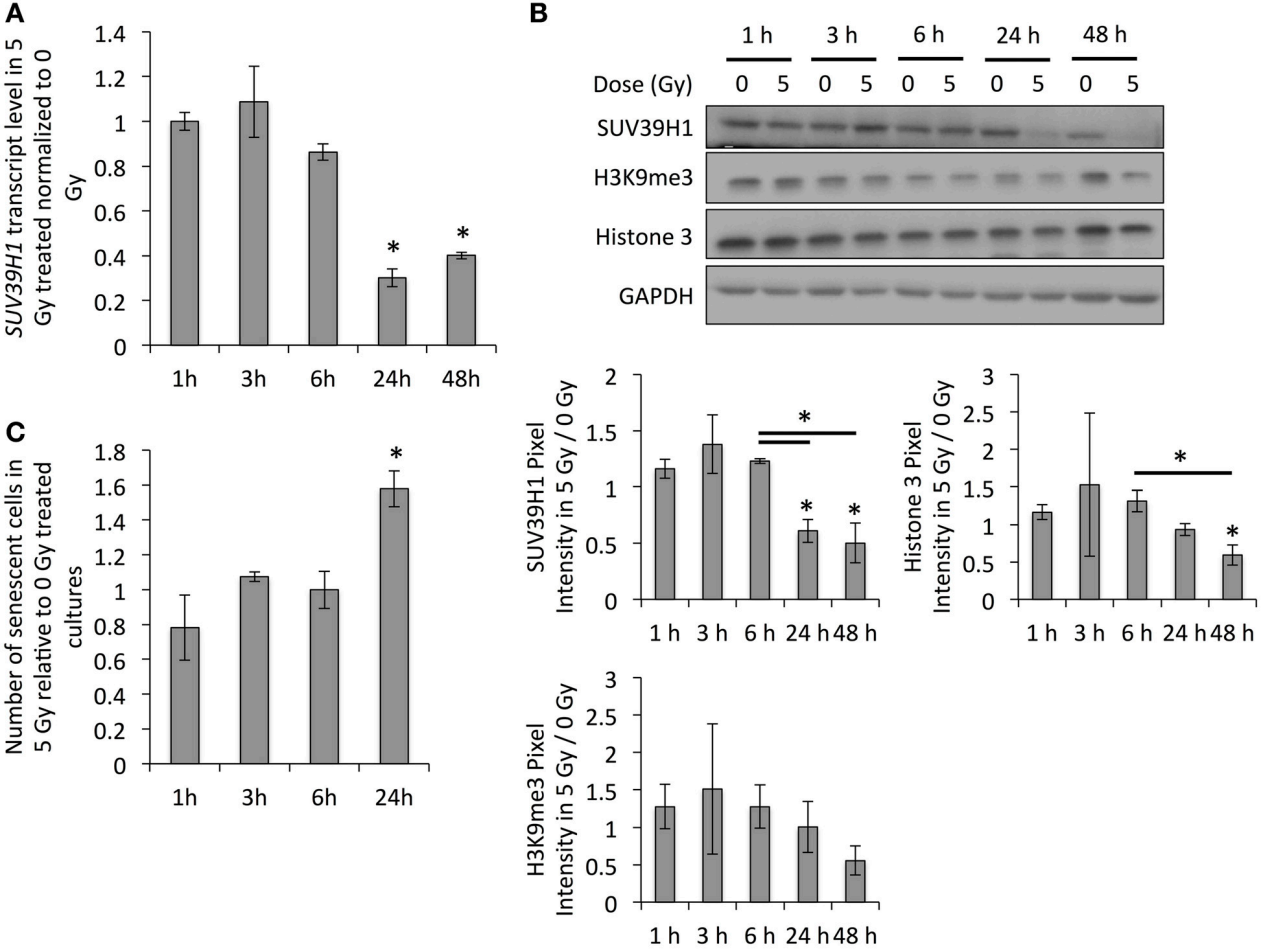
**SUV39H1 downregulation occurs during the first 24 h after exposure. (A)** *SUV39H1* transcript levels normalized to the expression level 1 h after exposure. Averages from three biological and two technical replicates per sample. Error bars indicate the error progression of the SDs. Arbitrary units, A.U. **(B)** Western blot images and quantifications. Averages of three biological replicates ± *SD*. **(C)** Fraction of senescent cells as determined by SA-β-GAL staining. Averages of three samples ± *SD*. ^*^p < 0.05.

As this downregulation of *SUV39H1* and H3K9me3 levels with increasing age or stage of senescence has been observed in multiple models (Scaffidi and Misteli, 2006; Sidler et al., 2013), it probably plays an important role in establishing senescence-related changes to the chromatin structure. If this downregulation of SUV39H1 plays a role in the establishment of radiation-induced senescence, it is expected to occur around the time that cells usually recover from the DNA damage checkpoint, which is between 12 and 24 h after exposure to irradiation (Xu et al., 2002). In order to test this, we examined *SUV39H1* transcript and protein levels and studied the induction of senescence based on SA-β-GAL activity in PD 38 cells exposed to 5 Gy at different time points after exposure to IR (Figure 5). Our results revealed that the *SUV39H1* transcript levels were slightly reduced at 6 h after exposure and were significantly decreased at 24 and 48 h after exposure to IR (Figure 5A), which corresponded to the reduced protein levels at the same time points (Figure 5B). This was associated with the trend of reduced H3K9me3 levels and the induction of senescence, which was significantly increased 24 h after exposure to 5 Gy (Figures 5B,C).

### SUV39H1 downregulation correlates with expression of satellite transcripts and DNA damage checkpoint

**Figure 6:**
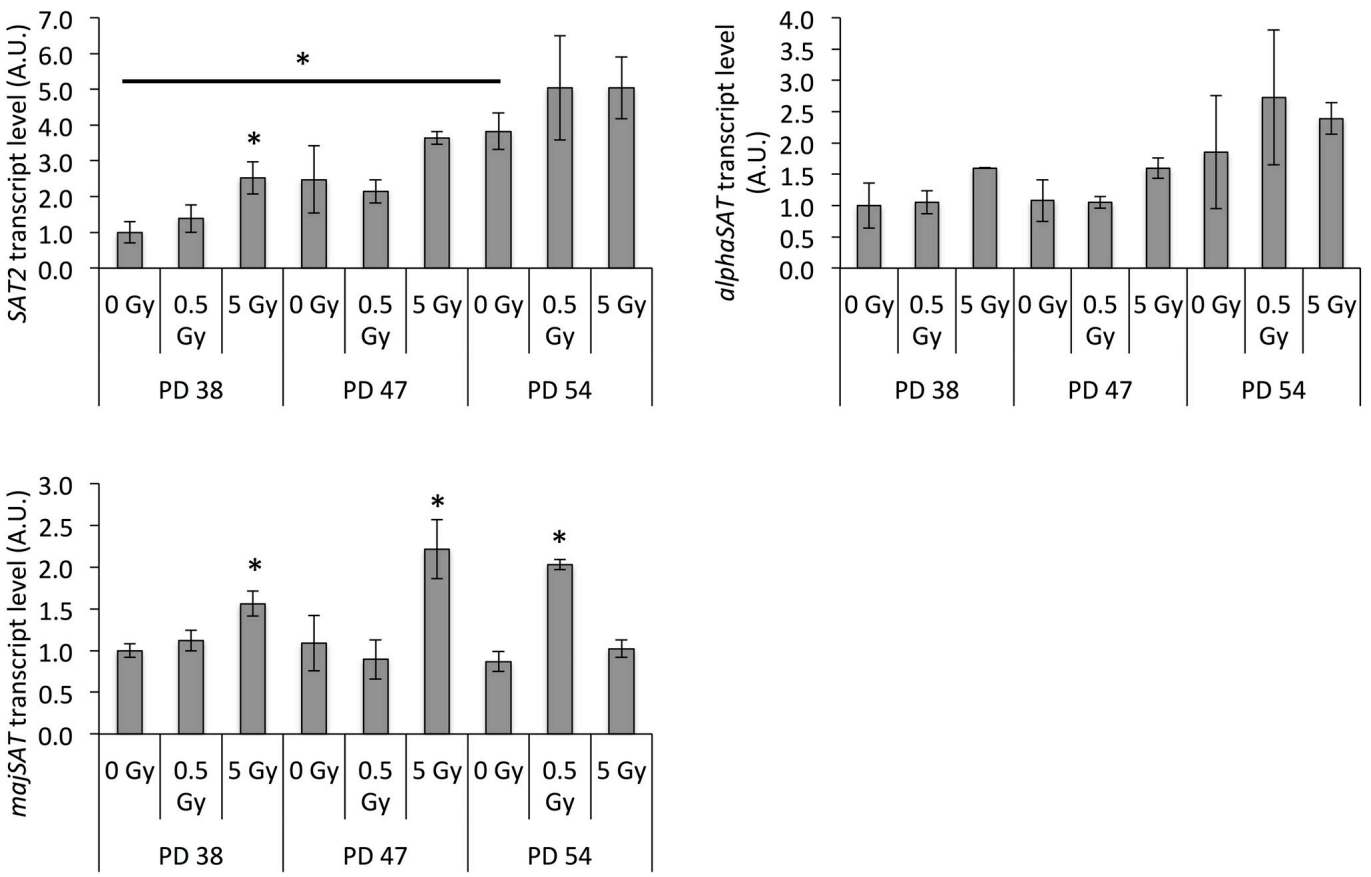
**Radiation exposure results in genomic instability**. Average transcript levels of satellite transcripts normalized to the expression in PD 38 0 Gy, shown as arbitrary units (A.U.). Error bars indicate the error progression of *SD*. ^*^p < 0.05.

SUV39H1 is important for pericentric heterochromatin formation and silencing of satellite regions (Peters et al., 2001; Lehnertz et al., 2003). Our previous results indicated that the senescence-associated downregulation of SUV39H1 resulted in the loss of H3K9me3 in satellite regions and increased the expression of satellite-derived transcripts (Sidler et al., 2014). Thus, we next tested whether the radiation-induced downregulation of SUV39H1 was associated with increased expression of satellite transcripts. Our results showed that the *SAT2* and *majSAT* transcripts were induced in IR-exposed PD 38 cells and PD 47 cells, although for PD 47 cells this induction was only significant for *majSAT* transcripts (Figure 6). Furthermore, both irradiated PD 38 and PD 47 cells exhibited a trend of increased *αSAT* expression (Figure 6). However, in PD 54 cells, only exposure to 0.5 Gy IR induced an increase in *majSAT* transcription (Figure 6).

**Figure 7:**
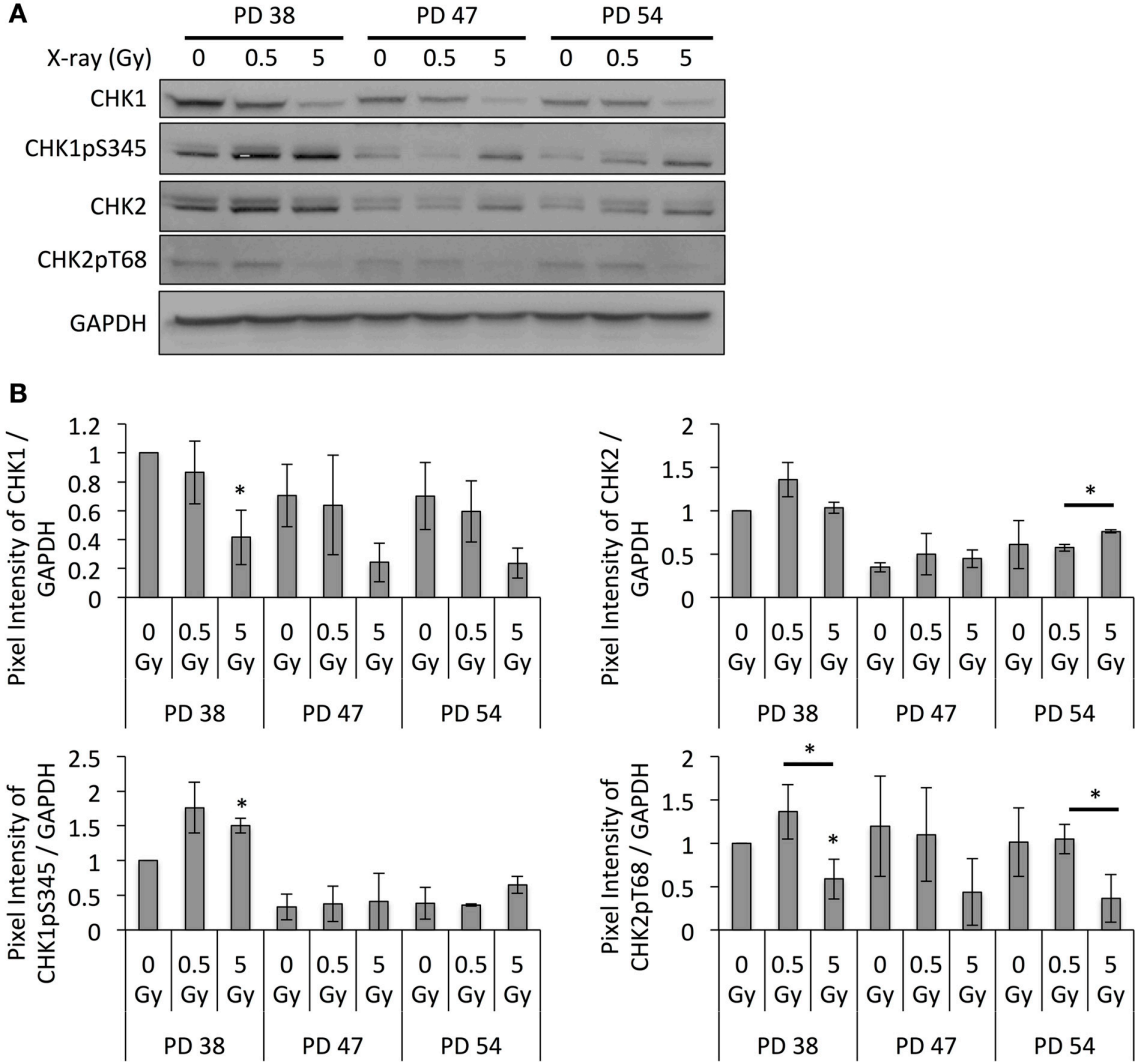
**Altered DNA damage checkpoint regulation. (A)** Representative Western blot images. **(B)** Western blot band quantifications of protein levels, normalized to PD 38 0 Gy. Averages from three biological replicates ± *SD*. ^*^p < 0.05.

In order to examine whether this increased chromatin relaxation and transcription of satellite regions correlated with genomic instability and DNA damage checkpoint activation, the protein levels of DNA damage checkpoint regulators were detected by Western blotting. Results revealed that exposure to increasing doses of X-ray irradiation resulted in the downregulation of CHK1 at all PD levels, while the remaining CHK1 proteins showed increased phosphorylation levels at Ser345 (Figure 7). The CHK2 protein and phosphorylation levels showed an opposite trend, where IR exposure resulted in a slight increase in the CHK2 protein levels and a reduction in the phosphorylation levels of Thr68 (Figure 7).

### SUV39H1 overexpression inhibits radiation-induced premature senescence

**Figure 8:**
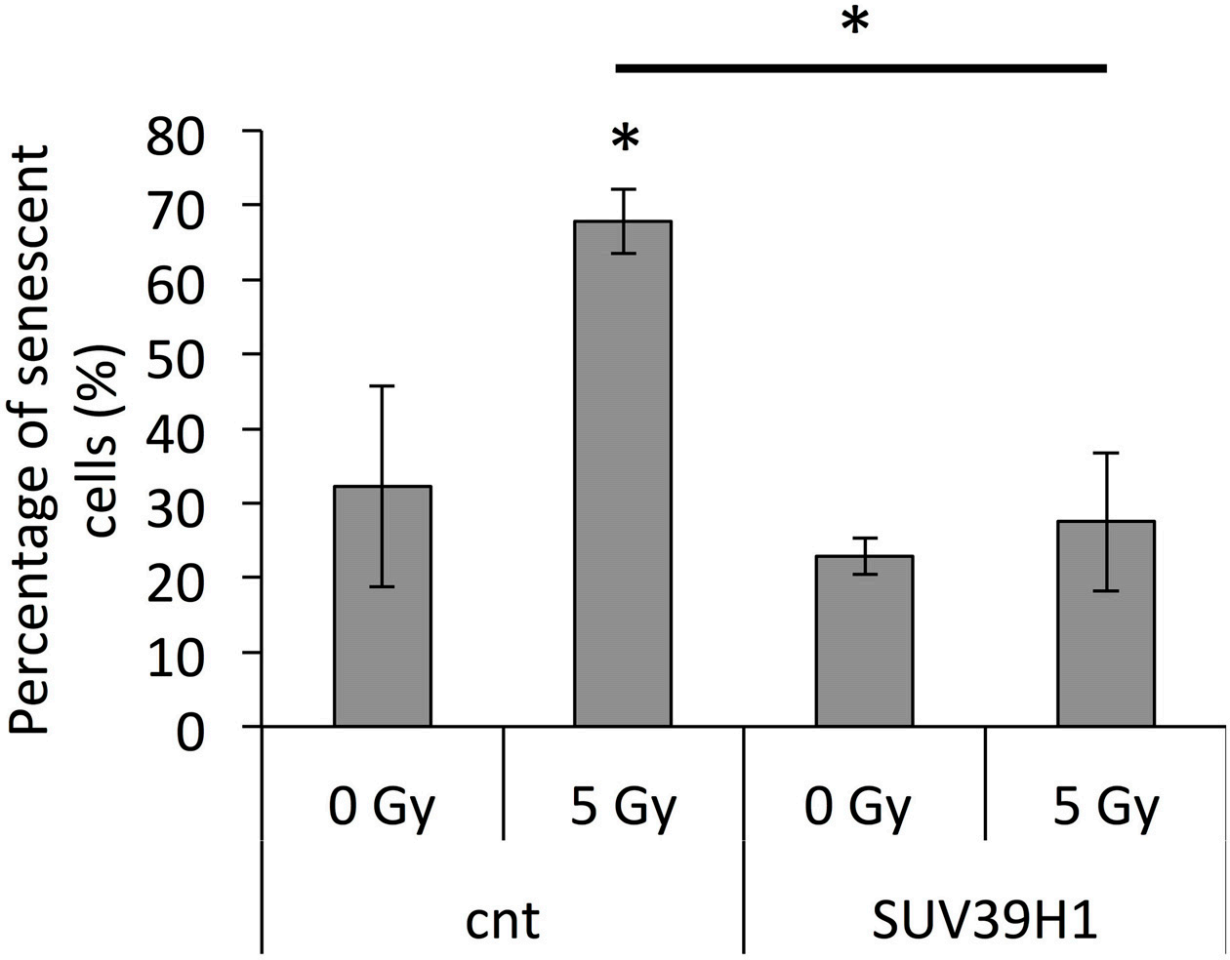
**SUV39H1 overexpression inhibits radiation-induced senescence**. Averages ± *SD* of senescent cells after radiation exposures, when cells had been pre-treated with an SUV39H1 overexpression vector. ^*^p < 0.05.

Our previous study showed that the inhibition of SUV39H1 resulted in the inhibition of cell division and the induction of senescence in pre-senescent cells (Sidler et al., 2014). The observed downregulation of SUV39H1, the reduction in H3K9me3 levels and the induction of satellite transcription after exposure to IR, resembled the previously described senescence-dependent changes. Thus, as a next step, the role of SUV39H1 in radiation-induced senescence was further examined by overexpressing *SUV39H1* in PD 38 cells prior to exposure to 5 Gy of X-ray. The overexpression of SUV39H1 before IR exposure prevented the IR-dependent induction of senescence (Figure 8).

### Binding of acetylated p53 to the SUV39H1 promoter is reduced after exposure to IR

Since the IR-dependent downregulation of SUV39H1 seemed to play an important role in the induction of senescence, the next step was to examine what might cause this downregulation. p53 was previously shown to indirectly downregulate *SUV39H1* expression through the p21-mediated suppression of E2F activity during the DNA damage response following IR, in order to allow for efficient repair of DNA double-strand breaks within heterochromatin regions (Zheng et al., 2014). In addition, MDM2, another p53 target gene, contributed to the decrease in SUV39H1 protein levels through inducing its proteasomal degradation (Mungamuri et al., 2012).

**Figure 9:**
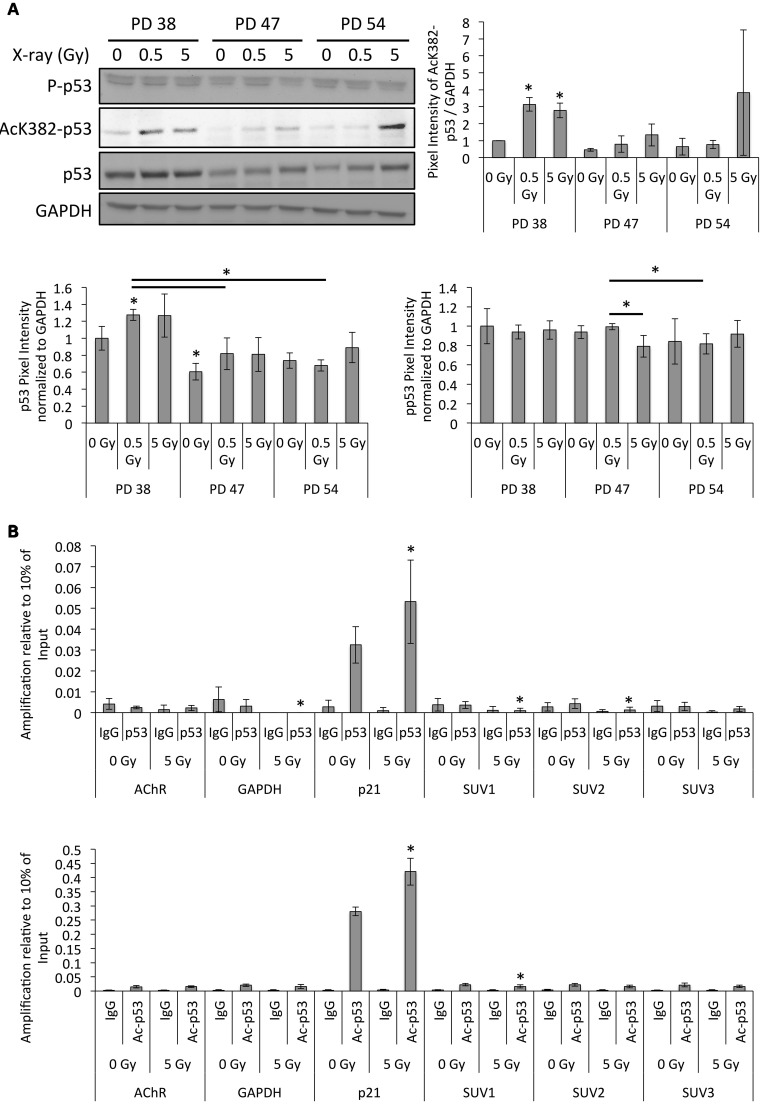
**Altered p53 activation and binding to the *SUV39H1* promoter after irradiation. (A)** Representative Western blots images and quantification of band intensities of the protein levels normalized to PD 38 0 Gy. Averages from three biological replicates ± *SD*. **(B)** ChIP-qRT-PCR target amplification normalized to 10% of the amplification from input DNA fragments. Averages from three biological replicates ± *SD*. ^*^p < 0.05.

In order to determine whether p53 plays a role in the regulation of SUV39H1 expression to mediate IR-induced senescence, we determined the protein levels of p53 as well as its phosphorylated and acetylated (Luo et al., 2004) active forms by Western blot analysis (Figure 9A). While the p53 protein levels were slightly induced in IR-exposed PD 38 and PD 47 cells, their phosphorylation levels remained almost unaffected (Figure 9A). However, acetylation at K382 of p53 significantly increased in IR-exposed PD 38 cells, and a similar trend was observed in the IR-exposed PD 47 and PD 54 cells (Figure 9A).

Since the acetylated form of p53 is active in transcriptional regulation (Luo et al., 2004), we were interested in testing whether it may directly regulate *SUV39H1* expression. To this end, we scanned the promoter region of *SUV39H1* for p53 transcription factor-binding sites (TFBS) using PROMO (Messeguer et al., 2002) and TFBIND (Tsunoda and Takagi, 1999) softwares. Results revealed that the promoter region contains several potential TFBS for p53.

In order to test whether p53 or acetylated p53 directly bind to the *SUV39H1* promoter and whether this was affected by exposure to IR, chromatin immunoprecipitation experiments using antibodies targeted to p53 or acetylated p53 were performed after exposure of PD 38 to 5 Gy. Since *p21* is a known target for transcriptional regulation by p53, amplification of the *p21* promoter region served as a positive control. Both p53 and acetylated p53 levels increased in the promoter regions of *p21* after exposure to 5 Gy (Figure 9B). The p53-dependent increase in the activation of *p21* transcription has been associated with the indirect inhibition of *SUV39H1* transcription. While the amplification of the *SUV39H1* promoter fragments was not significantly different when comparing the IgG negative control and p53 pulldown, it was significantly higher in the acetyl-p53 pulldown samples when compared to IgG controls (Figure 9B). Additionally, the amplification of the SUV1 amplicon was significantly reduced in 5 Gy when compared to the 0 Gy control samples (Figure 9B), suggesting that the binding of active, acetylated p53 to the *SUV39H1* promoter is reduced in response to IR.

### Changes in the DNA methylation patterns inversely correlate with the induction of senescence

Changes in the distribution of repressive histone marks were previously shown to correlate with changes in DNA methylation patterns in the affected genomic regions of senescing human mesenchymal stem cells (Schellenberg et al., 2011). On the other hand, IR is known to affect DNA methylation profiles as well. Several studies have shown that sequence-specific changes in DNA methylation occur after IR and persist through several cell divisions (Kovalchuk et al., 2004; Kaup et al., 2006). Thus, we hypothesized that IR may affect DNA methylation patterns in a senescence-dependent way.

**Figure 10:**
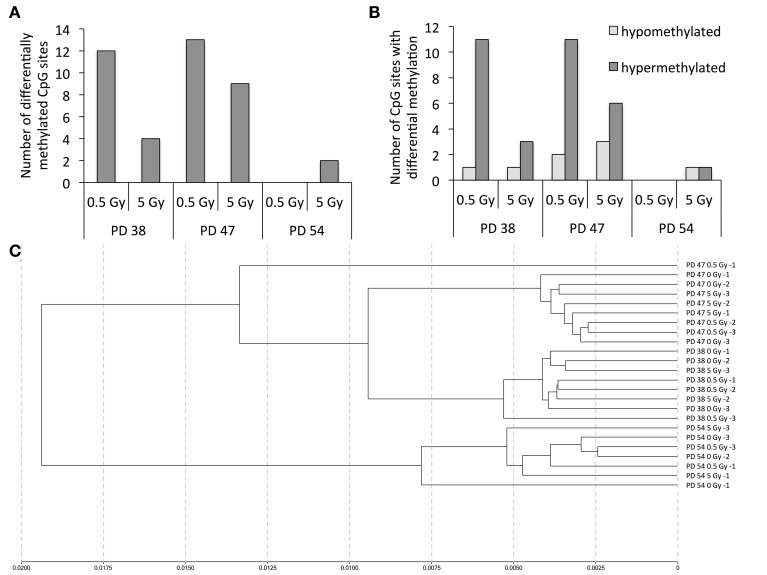
**Changes in the DNA methylation pattern in response to senescence and X-ray irradiation**. **(A)** Numbers of CpG sites affected by differential methylation when comparing cells of different passages exposed to different doses of X-ray irradiation. **(B)** Numbers of hyper- (dark gray) and hypo- (light gray) methylated CpG sites in irradiated pre-senescent and senescent cultures. **(C)** Cluster analysis.

To investigate this hypothesis, we profiled the CpG sites affected by changes in DNA methylation. The results revealed that treatment with 0.5 Gy induced a higher number of differentially methylated CpG sites than treatment with 5 Gy in the PD 38 and PD 47 cultures (Figure 10A, S1). Further, a higher number of CpG sites were affected by differential methylation in the younger cultures (PD 38 and PD 47) exposed to IR than in the older culture (PD 54). Among the observed changes in methylation, hypermethylation was more frequent than hypomethylation (Figure 10B), which is in line with previously reported observations (Kaup et al., 2006).

Interestingly, the clustering analysis revealed that samples clustered primarily based on the stage of senescence of the cultures rather than according to the dose of radiation received (Figure 10C), and only very few CpG sites were differentially methylated in more than one treatment group (Table 2). The changes in the methylation levels of those CpG sites did not correlate with changes in the gene expression of the genes in those regions. Thus, while such changes in methylation may enable cells to adapt to IR, the functional implications of these methylation changes remain to be determined.

**Table 2:** **CpG sites affected by change in methylation in more than one experimental group**.

		**PD 38**		**PD 47**		**PD 54**	
	**Genes in this region**	**0.5 Gy**	**5 Gy**	**0.5 Gy**	**5 Gy**	**0.5 Gy**	**5 Gy**
Chr1:110031996	GSTM1			−	−		
Chr9:113976778	SUSD1		^+^	^+^			^+^
Chr16:30841835	LOC283932			^+^	^+^		
Chr17:38531106	NBR2	^+^		^+^			
Chr19:10352577	TYK2			−	−		

+ *indicates hypermethylation, − indicates hypomethylation*.

## Discussion

Here, we showed that WI-38 cells with low senescence ratio display a distinct physiological response to IR when compared to cells with higher senescence ratio. While cell cycle arrest, apoptosis, and most prominently senescence was induced in response to IR in young cultures, old cultures showed an increase in cell death in response to IR but no significant induction of cell cycle arrest and only a slight increase in the percentage of senescent cells. The induction of senescence before the replicative limit is reached, also termed premature senescence, in response to IR has been previously described (Noda et al., 2012) and both senescence and apoptosis may contribute to several of the observed side effects of radiation therapy *in vivo*, such as tissue inflammation and fibrosis (Gallet et al., 2011), as well as developmental deficits in pediatric patients (Krasin et al., 2010).

To better understand the molecular changes associated with this increased senescence ratio in irradiated WI-38 cultures, we studied the changes in gene expression profiles, and epigenetic regulation. Cell cycle regulation-related genes comprised the largest fraction of downregulated genes in the 5 Gy-exposed groups at all PD states; this correlates with the observed changes in the cell cycle profiles in PD 38 and PD 47 and the induction of senescence. However, other functional categories, including epigenetic and transcriptional regulators, were also affected by exposure to 5 Gy.

Additionally, senescence has also been associated with changes to chromatin structure and epigenetic regulation. The observation that both SUV39H1, which specifically trimethylates H3K9 (Rea et al., 2000), and *CBX5*, which binds to H3K9me3 and mediates its repressive function (Bannister et al., 2001; Lachner et al., 2001), are downregulated in IR-treated PD 38 and PD 47 cells suggests that they may play a role in the response to IR. This downregulation of *SUV39H1* was confirmed at the RNA and protein levels and corresponded with reduced H3K9me3 levels.

In contrast with our observations, however, another study described an increase in the SUV39H1 and H3K9me3 protein levels during senescence or in response to genotoxic or oxidative stress (Bosch-Presegue et al., 2011). However, this may depend on the time points considered: while an increase in the SUV39H1 protein levels during the initial stress response or establishment of senescence may be important to limit genomic instability or adjust gene expression patterns such as by silencing the promoters of E2F target genes (Nielsen et al., 2001; Narita et al., 2003), the downregulation of SUV39H1 may be relevant for the maintenance of cell cycle arrest or senescence in the case of unsuccessful DNA repair.

The disruption of the human *SUV39H* genes *SUV39H1* and *SUV39H2* has been previously shown to induce a loss of H3K9me3 in constitutive heterochromatin regions, including the telomere regions (Garcia-Cao et al., 2004), thereby resulting in deheterochromatinization and genomic instability (Peters et al., 2001). In addition, *SUV39H1* has also been shown to act as a co-repressor to several transcription factors by its recruitment to and histone methylation in the promoter regions of specific genes (Nielsen et al., 2001; Jang et al., 2007; Cherrier et al., 2009; Wakabayashi et al., 2011; Mungamuri et al., 2012). Further, SUV39H is also required for chromatin condensation and mitotic progression (Melcher et al., 2000; Park et al., 2011). The observed downregulation of *SUV39H1* in response to IR may therefore result in G2/M arrest or deheterochromatinization resulting in genomic instability and stress-induced premature senescence.

We observed increased transcriptional abundance from satellite regions that are normally kept silent through SUV39H1-dependent H3K9 trimethylation, suggesting that the IR-induced downregulation of SUV39H1 induced the heterochromatin relaxation in the pericentric satellite regions. Our previous results showed such an induction of transcriptional activation of the pericentric satellite regions to be associated with the reduction of H3K9me3 levels in those regions during replicative senescence (Sidler et al., 2014), suggesting that the heterochromatin relaxation in those regions may contribute to genomic instability and growth arrest.

This transcriptional activation of the satellite regions correlated with the continued activation of CHK1, while the protein levels of CHK1 were reduced. CHK1 expression has been described to be cell cycle-dependent from the S to the M phases of the cell cycle (Kaneko et al., 1999), and CHK1 activation in response to DNA damage is responsible for long-term G2/M cell cycle arrest, as well as for cell cycle re-entry followed by apoptosis or G1 arrest and senescence, depending on the p53 and p21 status of the cell (Poehlmann et al., 2011). Thus, the observed continued phosphorylation of CHK1 may indicate that the cells exposed to X-ray irradiation are in a prolonged G2/M arrest 48 h post-exposure or have slipped into a G1 arrest. Interestingly, CHK1 has also previously been shown to be downregulated in response to DNA damage or ectopic p53 activation in a p21- and RB-dependent manner (Gottifredi et al., 2001). The same regulatory pathway was associated with the downregulation of SUV39H1 expression in response to DNA damage (Zheng et al., 2014). Thus, CHK1 and SUV39H1 may be co-regulated during the long-term DNA damage response and may mediate the slippage into the G1 phase of the cell cycle followed by senescence.

While previous studies have focussed on the effect of the differential expression of SUV39H1 on the induction of apoptosis (Mungamuri et al., 2012) or DNA repair (Zheng et al., 2014) in response to IR, we demonstrated that this altered expression may also play a role in the induction of senescence. Our previous results showed that *SUV39H1* overexpression in senescent cells induced cell division, while SUV39H1 inhibition in dividing cells slightly inhibited cell division (Sidler et al., 2014). The overexpression of *SUV39H1* prior to exposure to IR compensated for the radiation-induced downregulation of *SUV39H1*, preventing the induction of senescence.

The downregulation of SUV39H1 in response to IR also seems to be at least in part dependent on p53, since it is correlated with the induction of K382 acetylation of p53 and the reduction of acetylated p53 in the promoter region of *SUV39H1*. Together with the previous reports that showed the indirect effect of p53-mediated transcriptional regulation on the expression of SUV39H1 (Mungamuri et al., 2012; Zheng et al., 2014), these results may indicate a crucial role for this regulatory pathway in the long-term response to DNA damage.

Additionally, SUV39H1 and trimethylated H3K9 have been shown to recruit DNA methyltransferases at least in some sequence contexts and target them for DNA methylation (Fuks et al., 2003; Lehnertz et al., 2003). Thus, the reduced expression of SUV39H1, which correlates with the reduction in H3K9me3 levels, may also correlate with changes in DNA methylation.

Investigating the DNA methylation levels showed that IR induces changes in DNA methylation in the cells. In this case, younger cultures (PD 38 and PD 47) were more affected and exhibited a higher number of differentially methylated CpG sites in response to 0.5 Gy and 5 Gy. Further, site-specific CpG hypermethylation was observed more frequently than CpG hypomethylation, which is in line with previous observations (Kaup et al., 2006). As the Illumina® HumanMethylation27 BeadChip primarily contains probes that detect methylated CpGs in the promoter regions of genes, it enables the identification of changes in DNA methylation that are mainly considered to play roles in the regulation of gene expression. While the expression of the genes was not affected by the differential methylation in their promoter regions, these changes may have allowed the cells to adapt to the IR. Further, the observation that the DNA methylation profiles are similar between cultures of the same PD level regardless of the dose of radiation received may indicate that either the changes in IR-induced DNA methylation show a high variability between cells or that the potential DNA methylation changes induced by IR do not predominantly affect the CpGs within promoter regions.

On the other hand, the observation that the highest number of changes in DNA methylation was observed in 0.5 Gy-irradiated PD 38 and PD 47 cells, followed by 5 Gy-irradiated PD 38 and PD 47 cells may indicate that changes in CpG methylation following IR exposure may depend on cell division. Accordingly, the numbers of differentially methylated CpG sites detected in the different experimental groups roughly inversely correlated with the extent to which the cultures underwent IR-induced senescence. A recent study showed that human diploid fibroblasts and normal human bronchial epithelial cells that were exposed to ^137^Cs and did not undergo any further cell division after IR exposure did not show any significant changes in DNA methylation (Lahtz et al., 2012). Thus, senescent cells may be less susceptible to changes in DNA methylation in response to IR when compared to dividing cells.

In summary, we demonstrated that human diploid fibroblasts at three different stages of senescence showed different physiological responses to IR. While cultures with low and intermediate senescence ratios (PD 38 and PD 47) showed induction of G2/M cell cycle arrests, PD 38 and PD 54 cultures contained a higher percentage of dead cells 48 h after exposure to 5 Gy IR. More significantly, PD 38 and PD 47 cells exposed to IR underwent senescence. This was associated with changes in the expression of cell cycle regulators and senescence-associated genes, which may be modulated by changed expression or activity of several transcription factors. Further, *SUV39H1* as well as *CBX5*, an *HP1* homolog, were found to be downregulated in both PD 38 and PD 47 cells exposed to IR, and were correlated with reduced H3K9me3 levels and induced expression of transcripts from pericentric satellite regions. While such downregulation of SUV39H1 in response to DNA-damaging agents was previously associated with the induction of apoptosis or the facilitation of DNA repair, we suggest that this downregulation may also be associated with the induction of senescence in normal human diploid fibroblasts. This seems to at least in part depend on p53-dependent transcriptional suppression, suggesting that p53-mediated regulation of *SUV39H1* expression plays a role in the long-term DNA damage response. Within an organism, such an induction of senescence in dividing cells exposed to IR may interfere with the normal development and repair of a tissue or organ and thus contribute to side effects associated with IR.

### Conflict of interest statement

The authors declare that the research was conducted in the absence of any commercial or financial relationships that could be construed as a potential conflict of interest.
