# Supplementary material for: SUV39H1 downregulation induces deheterochromatinization of satellite regions and senescence after exposure to ionizing radiation
Source: Front Genet. 2014 Nov 21;5:411. doi: 10.3389/fgene.2014.00411 (PMC4240170; doi:10.3389/fgene.2014.00411)
Supplement: Supplementary file 1 [file Table1.DOCX]

**S1: Primers used for qRT-PCR.**

| Gene | Forward (F)/  Reverse (R) | Sequence | Annealing temperature | Reference |
| --- | --- | --- | --- | --- |
| *HPRT1* | F  R | 5ʹ-TGACACTGGCAAAACAATGCA-3ʹ  5ʹ-GGTCCTTTTCACCAGCAAGCT-3ʹ | 59.5°C | [1] |
| *YWHAZ* | F  R | 5ʹ-ACTTTTGGTACATTGTGGCTTCAA-3ʹ  5ʹ-CCGCCAGGACAAACCAGTAT-3ʹ | 59.5°C | [1] |
| *RPL13A* | F  R | 5ʹ-CCTGGAGGAGAAGAGGAAAGAGA-3ʹ  5ʹ-TTGAGGACCTCTGTGTATTTGTCAA-3ʹ | 59.5°C | [1] |
| *COL3A1* | F  R | 5ʹ-CGATGAGATTATGACTTC-3ʹ  5ʹ-ATTACAGAATACCTTGATAG-3ʹ | 53.0°C |  |
| *UBE2C* | F  R | 5ʹ-ACATATGCCTGGACATCCTGA-3ʹ  5ʹ-GGTTCTCCTAGAAGGCTCTGG-3ʹ | 59.5°C | [2] |
| *POT1* | F  R | 5ʹ-GGGCAAAGCAGAAGTGGACGGAGCATC-3ʹ  5ʹ-ATTGACAGATAACATCTGAATGCTGATTGGCTGTC -3ʹ | 59.5°C | [3] |
| *FOXM1* | F  R | 5ʹ-ACTTTAAGCACATTGCCAAGC-3ʹ  5ʹ-CGTGCAGGGAAAGGTTGT-3ʹ | 55.9°C | [4] |
| *E2F2* | F  R | 5ʹ-CCAAGAATTACATCAGAGAA-3ʹ  5ʹ-GCTTACATTCCAGACTTC-3ʹ | 55.9°C |  |
| *SUV39H1* | F  R | 5ʹ-CTACTATGGCAACATCTC-3ʹ  5ʹ-GTCAAGGTTGTCTATGAA-3ʹ | 55.9°C |  |
| *SAT2* | F  R | 5ʹ-CATCGAATGGAAATGAAAGGAGTC-3ʹ  5ʹ-ACCATTGGATGATTGCAGTCAA-3ʹ | 59.5°C | [5] |
| *majSAT* | F  R | 5ʹ-GACGACTTGAAAAATGACGAAATC-3ʹ  5ʹ-CATATTCCAGGTCCTTCAGTGTGC-3ʹ | 55.9°C | [5] |
| *alphaSAT* | F  R | 5ʹ-CTGCACTACCTGAAGAGGAC-3ʹ  5ʹ-GATGGTTCAACACTCTTACA-3ʹ | 55.9°C | [5] |

1. Vandesompele J, De Preter K, Pattyn F, Poppe B, Van Roy N, et al. (2002) Accurate normalization of real-time quantitative RT-PCR data by geometric averaging of multiple internal control genes. Genome Biol 3: RESEARCH0034.

2. Takahashi Y, Ishii Y, Nishida Y, Ikarashi M, Nagata T, et al. (2006) Detection of aberrations of ubiquitin-conjugating enzyme E2C gene (UBE2C) in advanced colon cancer with liver metastases by DNA microarray and two-color FISH. Cancer Genet Cytogenet 168: 30-35.

3. Baumann P, Podell E, Cech TR (2002) Human Pot1 (protection of telomeres) protein: cytolocalization, gene structure, and alternative splicing. Mol Cell Biol 22: 8079-8087.

4. Gemenetzidis E, Bose A, Riaz AM, Chaplin T, Young BD, et al. (2009) FOXM1 upregulation is an early event in human squamous cell carcinoma and it is enhanced by nicotine during malignant transformation. PLoS One 4: e4849.

5. Wang D, Zhou J, Liu X, Lu D, Shen C, et al. (2013) Methylation of SUV39H1 by SET7/9 results in heterochromatin relaxation and genome instability. Proc Natl Acad Sci U S A 110: 5516-5521.
