# Supplementary material for: SUV39H1 downregulation induces deheterochromatinization of satellite regions and senescence after exposure to ionizing radiation
Source: Front Genet. 2014 Nov 21;5:411. doi: 10.3389/fgene.2014.00411 (PMC4240170; doi:10.3389/fgene.2014.00411)
Supplement: Supplementary file 2 [file Table2.DOCX]

**S2: Antibodies used for Western blots.**

| Target | Supplier, Cat No | Dilution |
| --- | --- | --- |
| Mouse anti-SUV39H1 (m) | Abcam, ab12405 | 1:500 in 5% milk (PBST) |
| Rabbit anti-H3K9me3 (p) | Abcam, ab8898 | 1:500 in 5% milk (PBST) |
| Rabbit anti-H3K9ac (p) | Abcam, ab10812 | 1:500 in 5% milk (PBST) |
| Rabbit anti-H3 (p) | Cell Signaling, 9715 | 1:1000 in 5% milk (PBST) |
| Rabbit anti-CHK2 (p) | Abcam, ab8108 | 1:250 in 5% milk (PBST) |
| Rabbit anti-pT68CHK2 (p) | Abcam, ab3501 | 1:500 in 5% milk (PBST) |
| Mouse anti-CHK1 (m) | Cell Signaling, 2360 | 1:1000 in 5% BSA (PBST) |
| Rabbit anti-pS345CHK1 (p) | Cell Signaling, 2341 | 1:1000 in 5% milk (PBST) |
| Mouse anti-p53 (m) | Santa Cruz, sc126 | 1:1000 in 5% milk (PBST) |
| Rabbit anti-phospho p53 (p) | Santa Cruz, sc16716 | 1:250 in 5% milk (PBST) |
| Rabbit anti-acetylK382p53 (p) | Cell Signaling, 2525 | 1:250 in 5% BSA (PBST) |
| Rabbit anti-p21 (p) | Abcam, ab7960 | 1:1000 in 5% milk (PBST) |
| Mouse anti-GAPDH (m) | Santa Cruz, sc-47724 | 1:1000 in 5% milk (PBST |
| Secondary antibodies |  |  |
| Donkey anti-rabbit | Santa Cruz, sc2313 | 1:10000 in 5% milk (PBST) |
| Goat anti-Mouse | Santa Cruz, sc2005 | 1:5000 in 5% milk (PBST) |

PBST, phosphate-buffered saline-Tween 20; m, monoclonal; p, polyclonal
