# Supplementary material for: SUV39H1 downregulation induces deheterochromatinization of satellite regions and senescence after exposure to ionizing radiation
Source: Front Genet. 2014 Nov 21;5:411. doi: 10.3389/fgene.2014.00411 (PMC4240170; doi:10.3389/fgene.2014.00411)
Supplement: Supplementary file 3 [file Table3.DOCX]

**S4: Primers used for ChIP-qRT-PCR.**

| Target | F/  R | Sequence | Annealing temperature | Reference |
| --- | --- | --- | --- | --- |
| *AChR* | F  R | 5ʹ-CCTTCATTGGGATCACCACG-3ʹ  5ʹ-GGAGATGAGTACCAGCAGGTTG-3ʹ | 62.5°C | [[49](#_ENREF_49)] |
| *GAPDH* | F  R | 5ʹ-TACTAGCGGTTTTACGGGCG-3ʹ  5ʹ-TCGAACAGGAGGAGCAGAGAGCGA-3ʹ | 62.5°C | [[85](#_ENREF_85)] |
| *P21* | F  R | 5ʹ-CTGGACTGGGCACTCTTGTC-3ʹ  5ʹ-CCCTTCCTCACCTGAAAACA-3ʹ | 62.5°C | [[49](#_ENREF_49)] |
| *SUV1* | F  R | 5ʹ-ACTCTTGTGGGTTGGACAGG-3ʹ  5ʹ-GACAGACAGGAGCGAAGGTC-3ʹ | 62.5°C |  |
| *SUV2* | F  R | 5ʹ-GAGTGGGACACGGTAGCAAT-3ʹ  5ʹ-CATATCCAGGCTCTGCCATT-3ʹ | 62.5°C |  |
| *SUV3* | F  R | 5ʹ-GCAACTTGAGGACGTGACAG-3ʹ  5ʹ-CCAGCTGTGATTCCTGACAA-3ʹ | 62.5°C |  |
