# Supplementary material for: SUV39H1 downregulation induces deheterochromatinization of satellite regions and senescence after exposure to ionizing radiation
Source: Front Genet. 2014 Nov 21;5:411. doi: 10.3389/fgene.2014.00411 (PMC4240170; doi:10.3389/fgene.2014.00411)
Supplement: Supplementary file 6 [file Presentation2.PDF]

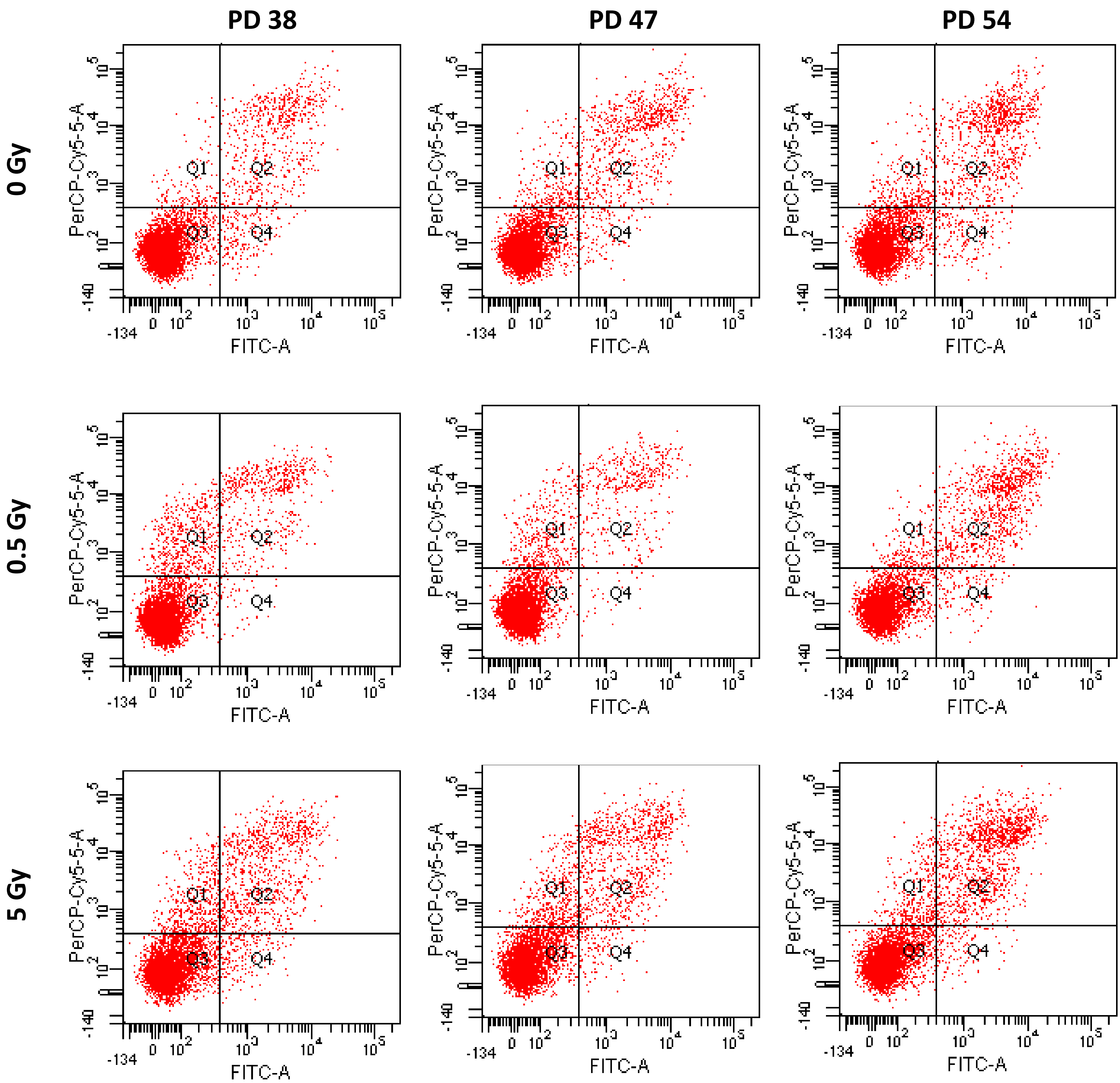

Representative Plots of flow cytometer analysis of Annexin V/PI staining.

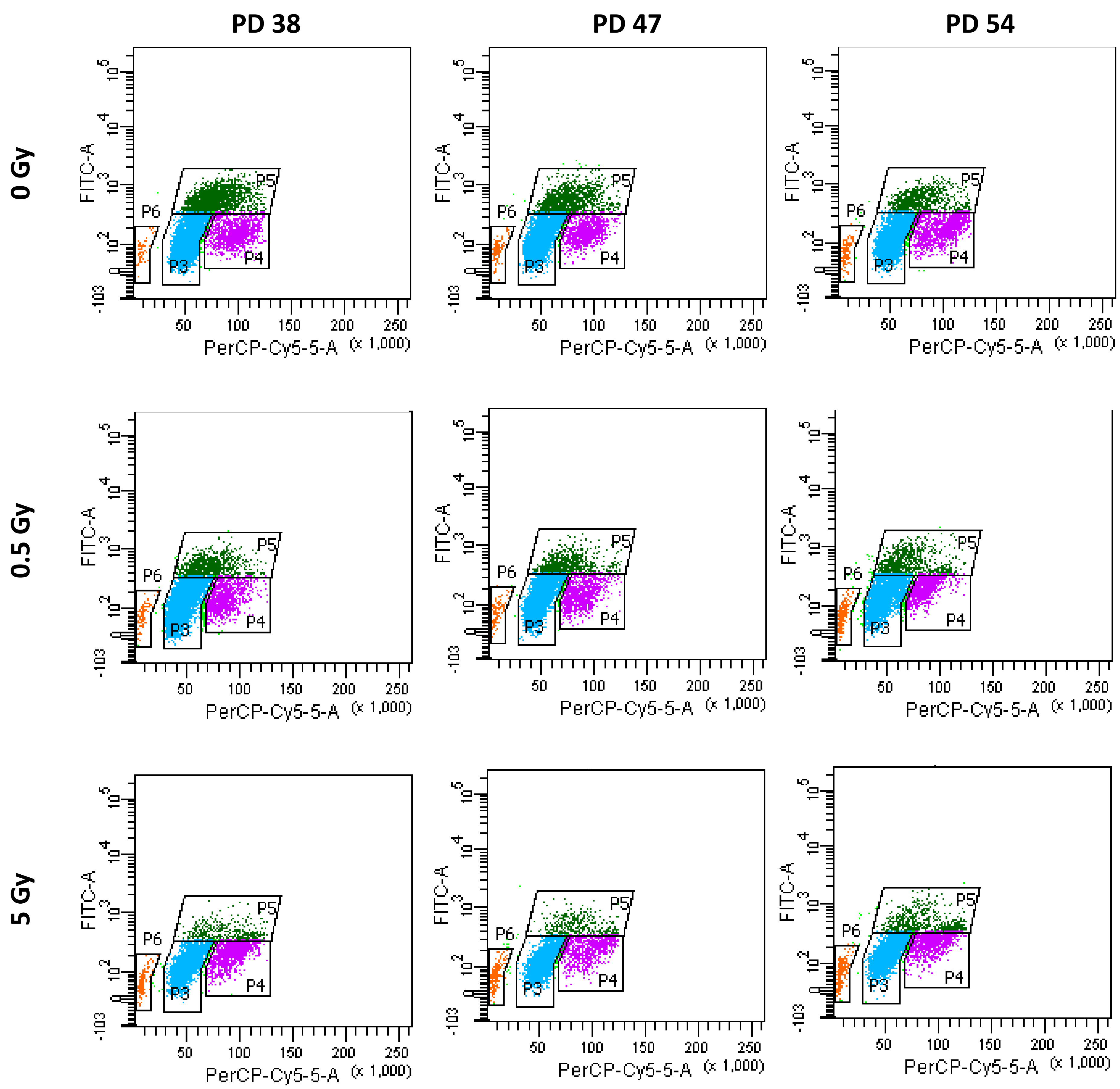

Representative Plots of flow cytometer analysis of BrdU staining. BrdU incorporation is detected by FITC, while the DNA amount was determined by PI staining (detected by PerCP-Cy5 channel).

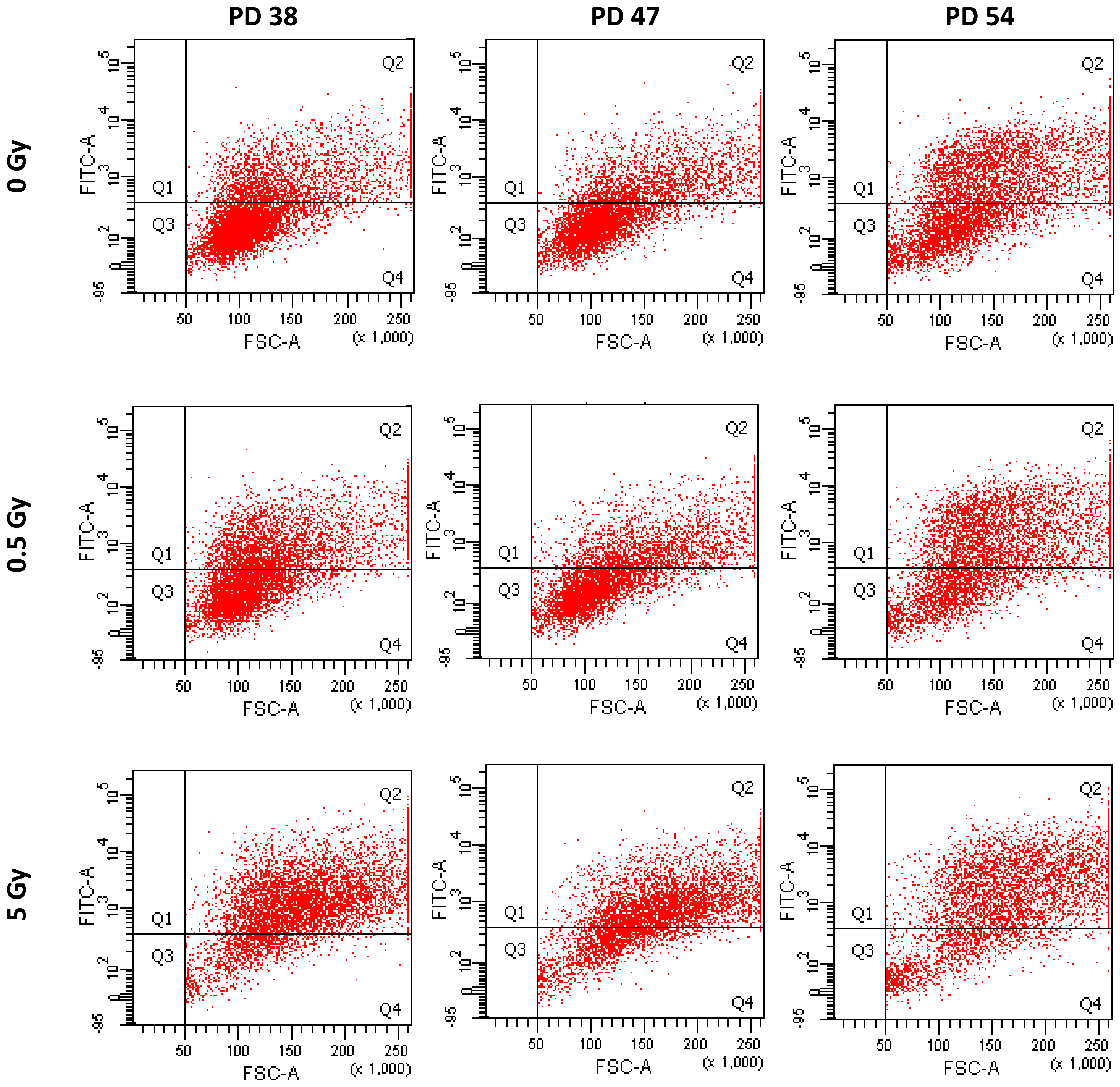

Representative Plots of flow cytometer analysis of senescence-associated β-Galactosidase staining. The FITC threshold was set based on a unstained control.
