# Supplementary material for: SUV39H1 downregulation induces deheterochromatinization of satellite regions and senescence after exposure to ionizing radiation
Source: Front Genet. 2014 Nov 21;5:411. doi: 10.3389/fgene.2014.00411 (PMC4240170; doi:10.3389/fgene.2014.00411)
Supplement: Supplementary file 7 [file Presentation3.PDF]

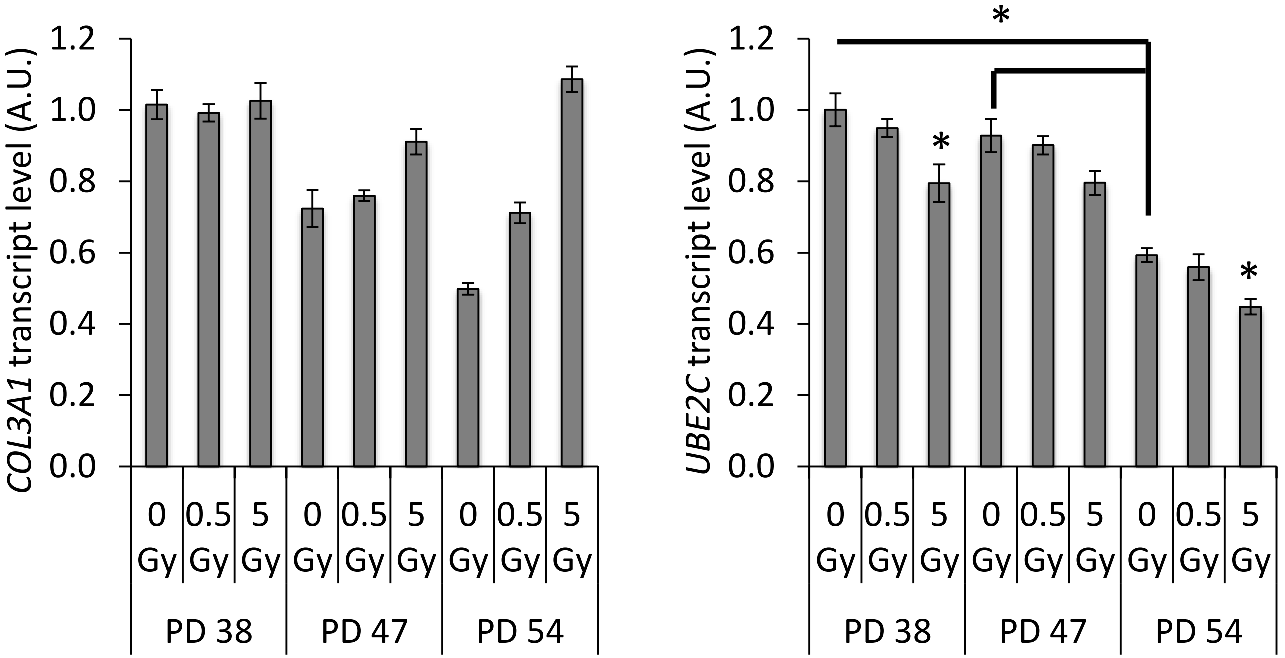

S5: Senescence-associated gene expression with increasing age and X-ray irradiation dose.

Transcriptional levels of *UBE2C*, and *COL3A1*. Averages from three samples and two technical repeats normalized to the expression in the control PD 38 cells, numbers are shown as arbitrary units (A.U.). Error bars indicate error progression of SDs.
